# Knowledge, attitude, and perception of energy drinks consumption among university students in Jordan

**DOI:** 10.1017/jns.2023.90

**Published:** 2023-11-03

**Authors:** Samar Thiab, Muna Barakat, Razan I. Nassar, Rana Abutaima, Asem Alsughaier, Roa'a Thaher, Faten Odeh, Wael Abu Dayyih

**Affiliations:** 1 Faculty of Pharmacy, Applied Science Private University, Amman, Jordan; 2 Faculty of Pharmacy, Zarqa Private University, Zarqa, Jordan; 3 Al-Balqa Applied University, Al-Salt, Jordan; 4 Faculty of Pharmacy, Mutah University, Al-Karak, Jordan

**Keywords:** Attitude, Energy drinks, Knowledge, Perception, University students

## Abstract

Energy drinks gained popularity after the launch of Red Bull in 1997. Different brands are now available and young adults mainly consume these drinks. This study assesses the knowledge, attitude, and perception of energy drink consumption among university students in Jordan. A validated online survey was used to collect the required data, extracted from Google Forms into an Excel spreadsheet and statistically analysed using Statistical Package for Social Sciences version 24.0. A nationally representative sample of university students with a mean age of 22⋅2 ± 3⋅9 years (*n* 749) was obtained. The participating students demonstrated a neutral level of knowledge about energy drinks, as the mean score of knowledge = 7⋅1 ± 2⋅2 (out of 12), with 66 % (*n* 498) of them having consumed energy drinks and experienced their effects. Generally, the study's participants demonstrated a neutral attitude towards energy drinks and 70⋅5 % (*n* 528) acknowledged that energy drinks increase activity, but more than 70 % of them believed that energy drinks have harmful side effects. It was found that there is a significant (*P*-value <0⋅5) positive correlation between knowledge score and female gender, studying a medical major, and monthly income. The main reasons for consuming energy drinks were reported to be: to stay awake for longer, help study, and become more energetic. There is a need for more structured awareness campaigns to warn students about the possible side effects of these products in order to reduce the consumption and popularity of these drinks among students.

## Introduction

Energy drinks gained popularity after the launch of Red Bull in 1997; afterwards, more than 500 different types of energy drinks were introduced until 2006.^(1,2)^ As the international market for energy drinks reached $15 trillion, its intake has risen accordingly, especially among university students and young people.^(3)^

Energy drinks are beverages that consist of a high level of caffeine, sugars, and other additives. Consequently, they become stimulants that can raise breathing rate, heart rate, and blood pressure.^(4)^ Along with the caffeine, energy drinks provide several ‘energy boosters’ including herbal extract (e.g. ginseng), amino acids and their derivatives (e.g. taurine and carnitine), and sugar derivatives such as ribose and glucuronolactone.^(1)^ Energy drink manufacturers implement marketing approaches claiming increased attention, alertness, concentration, and energy, targeting mainly young adults and teens.^(5)^

When consumed, energy drinks have an energising effect on adults between the ages of 18 and 55; this effect peaks 30–60 min after consumption and may last for no less than 90 min.^(6)^ One energy drink is approximately equivalent to 4–6 cups of coffee.^(7)^ Young adults and youths under the age of 35 make up more than half of the consumer market worldwide.^(8)^

For many reasons, university students take various types of energy drinks, unaware of the potential risks associated with them and have no idea where to turn for trustworthy information.^(3)^ However, in the quest to be at the top, educationally successful, the fastest, or the strongest, various students consume possibly damaging energy drinks.^(3)^ Students usually believe that energy drinks can improve attention during a long cognitive mental demand.^(3)^

Energy drinks campaigns across Jordan are well-known, and many universities participate and host such events. For example, the energy drink Boom Boom organised ‘All around Jordan City's Social Media Campaign’ in July 2021, where a giant can be pictured in several tourism sites in Jordan.^(9)^ Additionally, Red Bull organises several regular advertising and sports campaigns, such as Red Bull car park drift^(10)^ and Red Bull HATTRICK;^(11)^ both were also organised in 2022, in which many university students compete to participate increasing the widespread of these energy drinks consumptions among the youth population.

Limited research is done to estimate the prevalence, knowledge, attitude, and perception of young adults in Jordan regarding energy drinks. A study published in 2021 revealed that over half of the participating university students had poor knowledge about energy drinks and the prevalence of energy drinks consumption among them was 40⋅1 %.^(12)^ In another study, the prevalence of sugar-sweetened beverage consumption was found to be 60 %.^(13)^ This paper contributes to the literature by determining the proportion of university students who consume energy drinks, evaluating their knowledge, and assessing their perception while the energy drink advertising campaigns are increasing across Jordan.

## Experimental methods

### Study design and sample size

A cross-sectional online survey was carried out in Jordan from September to November 2022.

The required number of students to be recruited in this study was calculated according to the sample size equation for a cross-sectional survey^(14,15)^ based on the total number of students registered for the academic year 2020/2021 in Jordan, equating to 332 413 students.^(16)^ According to the equation used for calculating the sample size, the minimum sample size needed is 384 students to meet a 95 % confidence level (CI) and a 5 % margin of error.

The formula used to calculate the sample size is *n* = *Z*^2^⋅*p*⋅(1 − *p*)/*E*^2^, where *n* is the required sample size and *Z* is the *Z*-score corresponding to the desired CI. For a 95 % CI, *Z* is approximately 1⋅96; *p* is the population proportion; and *E* is the desired margin of error.

### Ethical approval

The study was approved by the Institutional/Local Research Ethics Committee, Amman, Jordan (Approval number: 2022-PHA-22).

### Survey development, validation, and data collection

Data collection was carried out using a self-administered online survey, which was developed using Google Forms and validated after extracting suitable questions from previous similar studies^(2,12,17–19)^ to obtain anonymous responses, which were treated confidentially. A brief description of the study and a consent statement was given at the beginning of the survey. Participation was voluntary, with no incentives offered. Eligible participants were any interested students enrolled in any university in Jordan. The participants were recruited through social media platforms: Facebook, WhatsApp, LinkedIn, and Twitter.

The survey was constructed in English but was delivered to the participants in Arabic. A panel of experts evaluated content and face validities. The first draft of the survey was evaluated by ten independent academics who have previous experience in this type of study and a statistician to assess the survey for appropriateness, complexity, attractiveness, and relevance of the items. The comments provided by the experts were taken into consideration and were incorporated as appropriate to the final version of the survey. The survey was then translated from English into Arabic and back by two bilingual senior academic staff members. The survey was then piloted on a sample of twenty-five academics and twenty-five non-academic people to enhance clarity, readability, and understandability and confirm its applicability to our targeted population.

The final version of the survey consisted of four sections that could be completed within 15 min (provided in the Supplementary material). The first section was designed to collect the participants’ demographic characteristics, while the second section was designed to assess the participants’ knowledge and source of information about energy drinks. Knowledge was assessed by giving 1 to the correct answer and 0 to the wrong answer. The scale measured knowledge from a maximum of 12 to a minimum of Zero. The score <2⋅4 were considered very unconfident, 2⋅4–4⋅8 as fairly unconfident, 4⋅9–7⋅2 as neutral, 7⋅3–9⋅6 as fairly confident, and 9⋅7–12 as very confident about the knowledge regarding energy drinks.^(20)^ The third section was designed to assess energy drinks consumption and experience among participants and the fourth and final sections were designed to assess the participants’ perception and attitude towards energy drinks. The attitude score was assessed using a Likert scale (strongly agree = 5, agree = 4, neutral = 3, disagree = 2, strongly agree = 1). The scale measured attitude from a maximum of 5 to a minimum of 1. Scores were categorised using Bloom's cut-off points, <3 (<59⋅0%) were considered a negative attitude, 3–3⋅9 (60⋅0–79⋅0 %) as neutral, and 4–5 (80⋅0–100⋅0 %) as a positive attitude towards energy drinks.^(21)^

### Statistical analyses

Data were extracted from Google Forms into an Excel spreadsheet and were then exported and statistically analysed using Statistical Package for Social Sciences version 24.0 (SPSS Inc., Chicago, IL, USA). Descriptive statistical analysis was used to analyse the socio-demographic data: mean and standard deviation (sd) for continuous variables. Categorical variables were demonstrated as frequencies and percentages. The Shapiro–Wilk test was used to assess the normality. Cronbach's *α* was used to evaluate the reliability of the questionnaire (=0⋅79), i.e. that the scales constructed are fit for their purpose, with values ≥0⋅7 indicating acceptable internal consistency. A point-biserial correlation was used to figure out whether there is an association between the score of knowledge and the dichotomous variables presenting the Pearson correlation and *P*-values. A *P*-value of <0⋅05 represents a significant difference.

## Results

### Demographic characteristics

A total of 749 surveys were completed and included in the final study analysis. The socio-demographic characteristics of the participants are demonstrated in Table 1. There was a predominance of male participants (*n* 495, representing 66⋅1 % of the sample) compared with females. The mean age of the respondents was 22⋅2 ± 3⋅9 years old and 695 were single. Most of the study sample expected to obtain a bachelor's degree (*n* 639, 85⋅3 %) and live in the middle of Jordan (*n* 585, 78⋅1 %), where the capital Amman is located. Around half of the respondent students were studying a major in the medical field (*n* 382, 51 %) and 66⋅2 % were studying in governmental universities (*n* 496). Around half of the participants’ family income was <750 Jordanian dinars; more than one-third were not medically insured (*n* 28, 137⋅5 %). Most of the participants (*n* 538, 71⋅8 %) were non-smokers of tobacco and (*n* 712, 95⋅1 %) did not have any chronic diseases.

**Table 1.** Socio-demographic characteristics of the study participants (*n* 749)

Variable	*n*	%
Gender		
Male	495	66⋅1
Female	254	33⋅9
Age (mean and sd) 22⋅2 ± 3⋅9		
Marital status		
Single	695	92⋅8
Married	48	6⋅4
Divorced	5	0⋅7
Widowed	1	0⋅1
University academic major		
Medical (medicine, pharmacy, nursing, medical tests, etc.)	382	51
Non-medical science major (engineering, computer science, information technology, basic sciences, etc.)	161	21⋅5
Sport pedagogy	5	0⋅7
Literature major (languages, law, business, humanities, etc.)	201	26⋅8
University sector		
Government	496	66⋅2
Private	253	33⋅8
Year of the study		
First year	97	13
Second year	131	17⋅5
Third year	147	19⋅6
Fourth year	198	26⋅4
Fifth year	128	17⋅1
Sixth year	48	6⋅4
Academic certificate expected to be obtained		
Diploma	27	3⋅6
BSc	639	85⋅3
Postgraduate studies (higher diploma, MSc, PhD)	83	11⋅1
Permanent place of residence in Jordan		
North Jordan	39	5⋅2
Middle Jordan (including the capital: Amman)	585	78⋅1
South Jordan	125	16⋅7
Family income in Jordanian dinars		
<250 JOD	73	9⋅7
251–500 JOD	168	22⋅4
501–750 JOD	138	18⋅4
751–1000 JOD	150	20
>1000 JOD	220	29⋅4
Health insurance		
Insured	468	62⋅5
Not insured	281	37⋅5
Are you a smoker of any tobacco products?		
Yes	211	28⋅2
No	538	71⋅8
Do you suffer from any chronic diseases?		
Yes	37	4⋅9
No	712	95⋅1

### Participants’ knowledge about energy drinks

The participants demonstrated a neutral level of knowledge about energy drinks, as the mean knowledge score = 7⋅1 ± 2⋅2 (out of 12). As shown in Table 2, the majority of the students knew that energy drinks contain caffeine (*n* 612, 81⋅7 %), and some may also contain herbal supplements and amino acids (86⋅4 and 87⋅6 %, respectively). Less than half of the participants knew that energy drinks contain L-carnitine, which increases fat burning (*n* 145, 19⋅4 %) and contains vitamins and minerals (*n* 292, 39⋅0 %). To a lesser extent, a minority of the participants knew that energy drinks could cause medical problems such as sleep problems (*n* 191, 25⋅5 %), headache (*n* 240, 32⋅0 %), and increase a person's impulsive behaviour (*n* 290, 38⋅7 %).

**Table 2.** Participants’ knowledge about energy drinks (*n* 749)

Statements	Correct answer^a^	
	*n*	%
Energy drinks contain caffeine.	612	81⋅7
Some energy drinks contain more sugar than the normal quota per person per day.	693	92⋅5
None of the energy drinks contain herbal preparations.	647	86⋅4
None of the energy drinks contain amino acids.	656	87⋅6
Some energy drinks contain L-carnitine, which increases fat burning.	145	19⋅4
Some energy drinks contain vitamins and minerals.	292	39⋅0
Some energy drinks contain animal-derived products.	630	84⋅1
Energy drinks don't cause sleep problems.	191	25⋅5
Energy drinks can cause digestive problems.	497	66⋅4
Energy drinks can cause a rapid heartbeat.	398	53⋅1
Energy drinks can't cause headaches.	240	32⋅0
Energy drinks cannot increase a person's impulsive behaviour.	290	38⋅7
**Knowledge score (mean ± sd)**	**7⋅1 ± 2⋅2**	

a Knowledge was assessed by giving 1 to the correct answer and 0 to the wrong answer. The scale measured knowledge from a maximum of 12 to a minimum of Zero. The score <2⋅4 were considered very unconfident, 2⋅4–4⋅8 as fairly unconfident, 4⋅9–7⋅2 as neutral, 7⋅3–9⋅6 as fairly confident, and 9⋅7–12 as very confident knowledge about energy drinks.

**Fig. 1.**
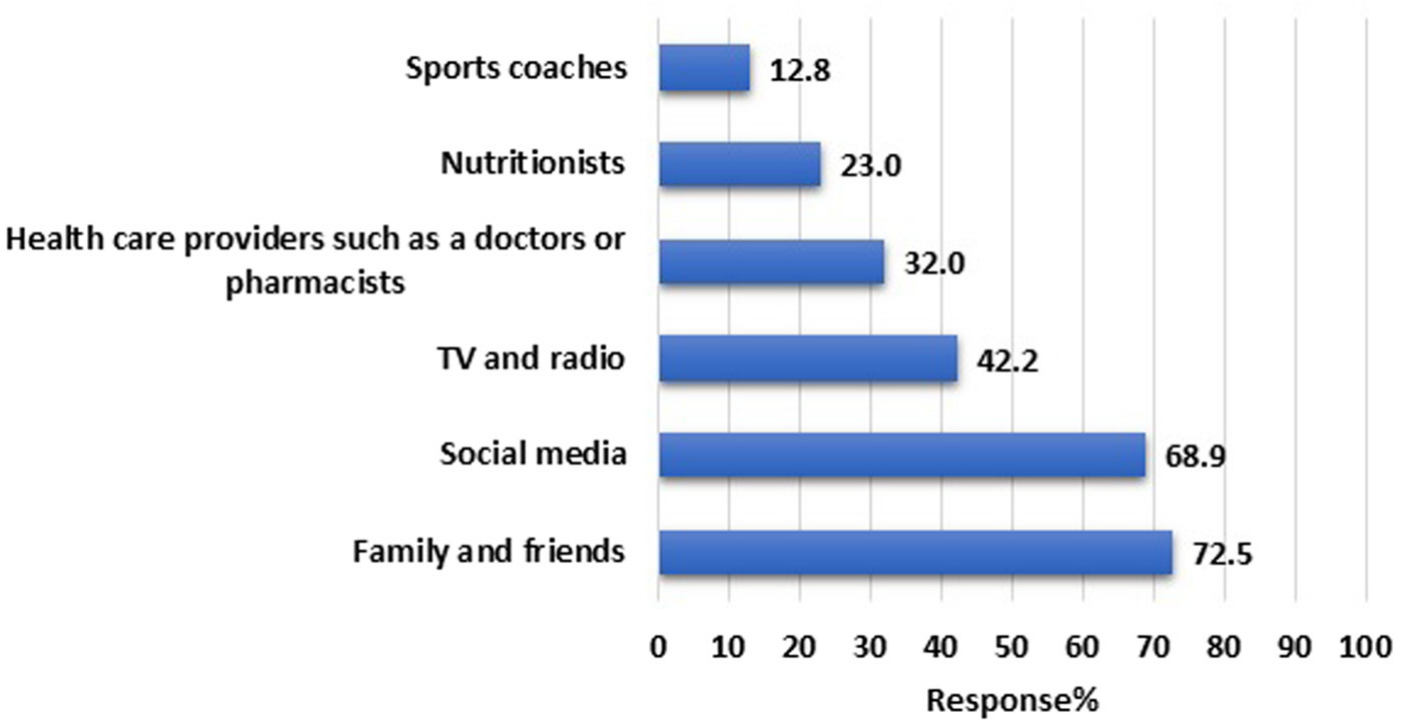
Sources of information about energy drinks among the study participants (*n* 749).

Fig. 1 demonstrates the main sources of information about energy drinks reported by the participants. The most common sources are family and friends (*n* 543, 72⋅5 %), followed by social media (*n* 516, 68⋅9 %) and television/radio (*n* 316, 42⋅2 %). While the role of healthcare providers was only 32⋅0 % (*n* 240), followed by nutritionists (*n* 172, 23⋅0 %) and sports coaches (*n* 96, 12⋅8 %).

### Energy drinks consumption and experience

**Fig. 2.**
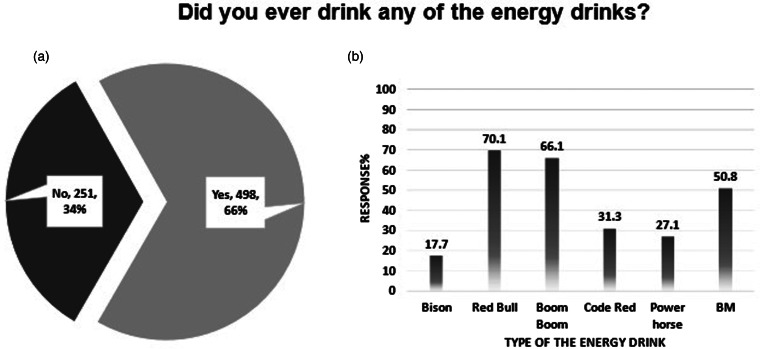
Participants reported usage of energy drinks. (a) Participants answers upon asking them if they drink these energy products. (b) Among the energy drinks users (*n* 498), this graph demonstrates the most common types of the used energy drinks.

Among the total sample size of the study, 66 % (*n* 498) of them have consumed energy drinks and experienced their effects, as shown in Fig. 2a. The majority of the participants of this category (energy drinks consumers) stated, in Fig. 2a, the most common products: Red Bull^®^, Boom Boom^®^, and BM^®^, 70⋅1, 66⋅1, and 50⋅8 %, respectively. Upon asking them about their weekly consumption of energy drinks, more than half of the users (*n* 275, 55⋅2 %) only drink one can per week. While around 15 % (*n* 78) were consuming more than five cans per week. Regarding the reasons for drinking energy products, as shown in Table 3, most of them declared that energy drinks help them to stay awake for a longer time (*n* 312, 62⋅7 %), help them to study for a longer time and accomplish tasks such as homework (*n* 309, 62⋅7 %), and to feel strong and energetic in general (*n* 269, 54⋅0 %). On the other hand, more than 90 % of them do not drink these energy products concomitantly with medications (*n* 475, 95⋅4 %), vitamins (*n* 487, 97⋅8 %), other herbs or natural products (*n* 474, 95⋅2 %), or alcohol (*n* 476, 95⋅6 %).

**Table 3.** Participants’ experience and causes for the use of energy drinks (*n* 498)

Question	*n*	%
How many cans of energy drinks do you use per week?		
Only one can	275	55⋅2
Two to three cans	90	18⋅1
Four to five cans	55	11⋅0
More than five cans	78	15⋅7
What is the reason that drives you to drink energy drinks? (more than one option was allowed)		
It helps me study for a longer time and accomplish tasks such as homework	309	62⋅0
It helps me focus and thus get better grades	198	39⋅8
It helps me feel better and reduces stress, fatigue, and exhaustion	180	36⋅1
To stay awake for a longer time	312	62⋅7
To feel strong and energetic in general	269	54⋅0
Other reasons	226	45⋅4
Have you taken an energy drink with medications such as painkillers or cough and cold remedies?		
Yes	23	4⋅6
No	475	95⋅4
Have you ever mixed an energy drink with vitamins?		
Yes	11	2⋅2
No	487	97⋅8
Have you ever mixed energy drinks with other herbs or natural products?		
Yes	24	4⋅8
No	474	95⋅2
Have you ever mixed energy drink with alcoholic drinks?		
Yes	22	4⋅4
No	476	95⋅6

### Perception and attitude towards energy drinks

**Fig. 3.**
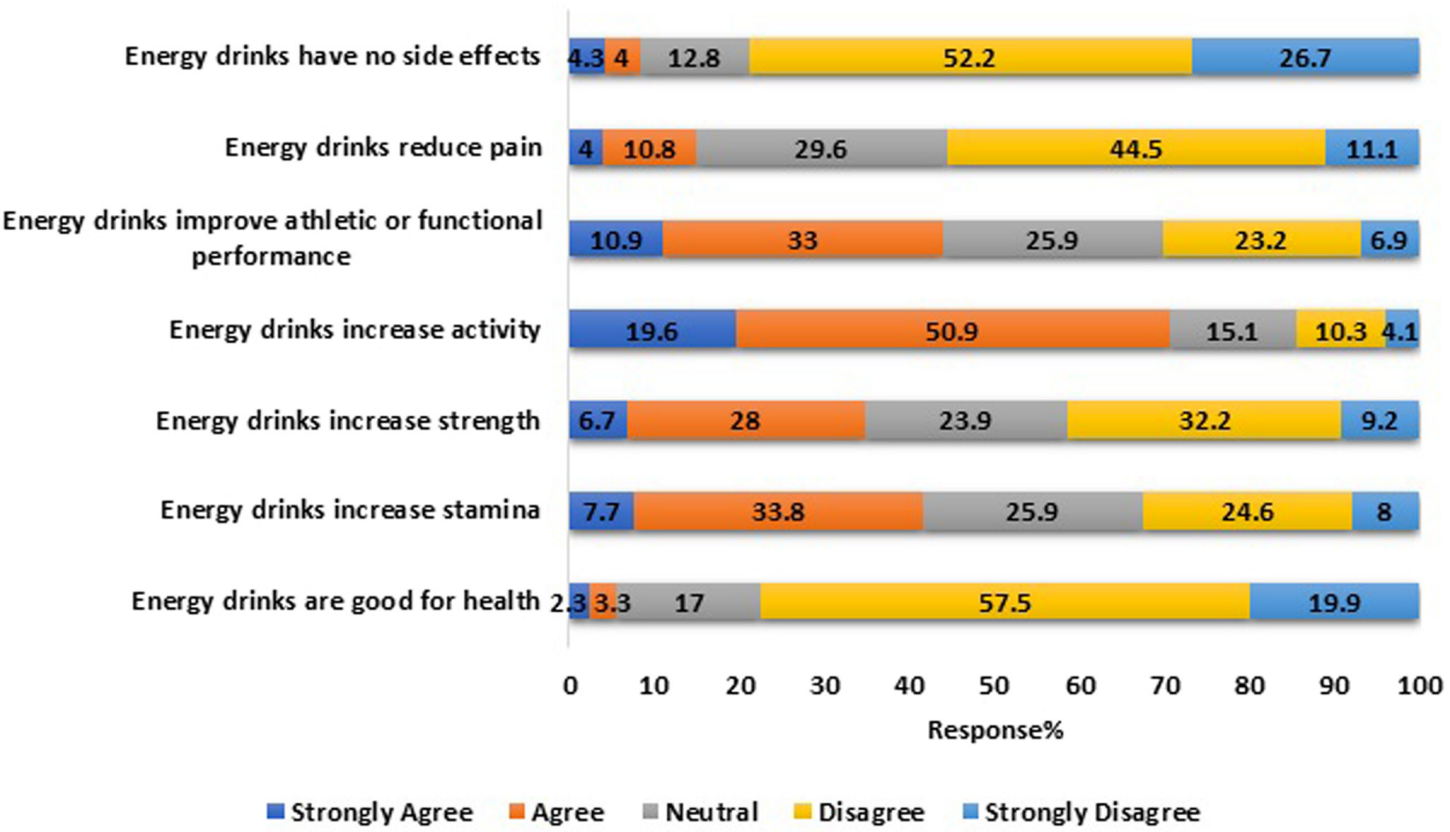
Participants’ perception towards energy drinks (*n* 749). Attitude score (mean ± sd) 3⋅9 ± 1⋅0. The attitude score was assessed using a Likert scale from a maximum of 5 to a minimum of 1.

In general, the study's participants demonstrated a neutral attitude towards energy drinks, as shown in Fig. 3. The majority of the students acknowledged (strongly agreed/agreed) that energy drinks increase activity (*n* 528, 70⋅5 %), and improve athletic or functional performance (*n* 329, 43⋅9 %), and stamina (*n* 311, 41⋅5 %). On the other hand, more than 70 % of the participants strongly disagree/disagree that energy drinks are good for health (*n* 580, 77⋅4 %) and have no side effects (*n* 591, 78⋅9 %).

Regarding the correlation between the knowledge score and the study variables, Table 4 shows a significant (*P*-value <0⋅5) positive correlation between knowledge score and female gender, education in medical subjects, academic years, and monthly income.

**Table 4.** Point-biserial correlation between score of knowledge about energy drinks and the dichotomous demographic variables

Variables	Knowledge score	
	Pearson correlation	*P*-value*
• Age	0⋅024	0⋅512
• Gender: Female *v.* male	0⋅171	**0⋅025**
• Marital status: Unmarried *v.* married	0⋅005	0⋅891
• Residential area: Urban *v.* rural	0⋅004	0⋅92
• University sector: Government *v.* private	0⋅056	0⋅126
• University academic major: Non-medical *v.* medical	−0⋅155	**<0⋅001**
• Educational level: University degree *v.* non-University degree	−0⋅029	0⋅429
• Academic year: >third year *v.* ≤third	0⋅164	**0⋅03**
• Monthly income: >500 *v.* <500 JOD	0⋅162	**0⋅04**
• Health insurance: Insured *v.* not insured	0⋅059	0⋅108
• Smoking status: smoker *v.* non-smoker	−0⋅045	0⋅222
• History of chronic diseases: Yes *v.* No	−0⋅004	0⋅915
• Attitude score	−0⋅022	0⋅542

* Significance measure at *P*-value <0⋅05 is presented in bold using point-biserial correlation.

## Discussion

This study is one of two studies conducted in Jordan to assess the prevalence, knowledge, and perception of the use of energy drinks among university students.^(12)^ This study is the only one conducted after many structured campaigns organised by several energy drink manufacturers in which the use of energy drinks was linked with sports activities such as football and drifting.^(10,11)^ These activities are attractive to university students, particularly males. This can also explain why more male students are interested in filling out this survey than female students. Similar behaviour was also observed in studies conducted in different countries, including Zambia,^(22)^ Taiwan,^(23)^ Poland,^(24)^ Saudi Arabia,^(25)^ the United Arab Emirates,^(26)^ and Lebanonv^(17)^ as well as in the only study conducted in Jordan in 2020,^(12)^ where it was noted that males are more daring and willing to try new things when compared to females.

Regarding the participating students’ knowledge about energy drinks, the majority knew that these drinks contain caffeine and sugars and may also contain amino acids and herbal extracts. However, many were unaware that these drinks might also contain L-carnitine, vitamins, and minerals, indicating that students do not generally check the constituents of the products they consume. Additionally, only around one-third of the participants knew that energy drinks may cause sleep problems and headaches. Many of the energy drinks side effects are due to the fact that they contain a considerable amount of caffeine. Eight ounces of energy drinks typically have 80–141 mg of caffeine.^(2)^ The biological mechanism of caffeine is not fully realised yet; it positively impacts performance by reducing feelings of exhaustion, enhancing physical endurance, and increasing central drive.^(27)^ Although caffeine has some positive effects on mood and cognition at low doses (12⋅5–100 mg),^(28)^ it also has some harmful negative effects on health, including the promotion of diuresis,^(29)^ reduction of insulin sensitivity,^(30)^ disturbance of regular sleep patterns,^(3)^ increase in mean arterial blood pressure,^(31)^ and chronic daily headache.^(32)^ Studies conducted in Zambia and the United Arab Emirates among university students found that there was a statistically significant association between energy drink consumption and poor sleep quality.^(22,26)^

In the study conducted in 2020 by Elsahoury *et al.* in Jordan, it was found that more than 60 % of the participating students had poor knowledge regarding energy drinks. In this study, the participants had a neutral knowledge, which could be attributed to the fact that more organised energy drinks campaigns were organised in 2021 and 2022, which may have influenced them to get more information about these drinks. The poor knowledge about energy drinks’ ingredients and detrimental health effects was also reported in a similar study conducted in Saudi Arabia.^(3)^ On the other hand, in a study conducted in Poland, students had high knowledge about energy drinks and their side effects, yet, their knowledge was not reflected in their consumption of such drinks.^(24)^ A better understanding of energy drinks constituents and side effects was also observed among medical students in a study conducted in Türkiye.^(33)^

Friends and advertisements on various platforms were an important source of information regarding energy drinks for the Jordan students, as observed in this study and the one conducted in 2020.^(12)^ Energy drinks advertisements usually have persuasive cues like sports and celebrities, which can influence young adults and persuade them to consume these drinks.^(34)^ The strong influence of advertisement was also observed in a study conducted in the United Arab Emirates.^(18)^

Regarding the prevalence of energy drinks consumptions among the participating students, it was found that 66 % of the participants consumed energy drinks. This percentage is higher than what was found in a similar previous study that was conducted in 2020 in Jordan, where 40⋅1 % of the students who participated in that study reported the consumption of energy drinks.^(12)^ This obtained result of a high prevalence of energy drinks consumption among students in Jordan is consistent with similar studies conducted worldwide, as it has been reported that energy drinks consumption prevalence was around 50 % among students in Australia,^(19)^ Italy,^(35)^ the United States,^(2)^ and Canada^(36)^ (48, 56⋅2, and 63 %, respectively). Similar research conducted in the Middle East and North Africa (MENA) region showed that energy drinks are consumed by 42⋅7 % of Iraqis, particularly young adults,^(37)^ 52⋅6 % of Saudi Arabian medical students,^(38)^ and 63⋅6 % of Lebanese students.^(17)^ A higher percentage was reported in the United Arab Emirates, as more than 90 % of the students reported using energy drinks.^(18)^

Red Bull^®^ and Boom Boom^®^ were the most common brands of energy drinks used by the students who participated in this study. These two brands were also very popular among students in Lebanon.^(17)^ Red Bull was also reported to be the most favourite energy drink among young adults and students in Saudi Arabia^(3,25)^ and the United Arab Emirates.^(18)^ The students’ tastes in Zambia differed as their preferred energy drinks were Dragon^®^ and Wild Cat^®^.^(22)^

Most of this study's participants declared that they consume energy drinks to help them stay awake, study, and feel energetic. These mentioned reasons for using energy drinks were also mentioned by young adults who participated in similar studies that were conducted in Saudi Arabia,^(3,25,38)^ Lebanon,^(17)^ the United States,^(2)^ Poland,^(24)^ Zambia,^(22)^ and Taiwan.^(23)^

Interestingly, less than 5 % of the students who participated in this study reported mixing energy drinks with drugs or alcohol. However, in other similar studies conducted in Lebanon^(17)^ and the United States,^(2)^ around 50 % of the participants reported using alcoholic energy drinks. Mixing energy drinks with alcohol was also reported in Taiwan^(23)^ and Türkiye.^(33)^ In Poland, energy drinks were found to be commonly mixed with coffee or alcohol.^(24)^ A study conducted in 2009 and 2010 among university students in an urban setting found that energy drinks consumption was significantly related to drinking alcohol.^(39)^ In addition, energy drinks were associated with people committing more risky behaviours.^(40,41)^

More than two-thirds of this study's participants believed that energy drinks are harmful to health and have side effects but agreed that they increase physical activity. It was found in various studies that young people knew energy drinks have side effects and experienced some of them, yet they still consumed them for various reasons.^(2,17,24–26,33,38)^

This study is the first in Jordan to find a correlation between the knowledge of student participants regarding energy drinks and their demographics. In this study, female gender, medical education, and monthly income had a positive effect on the knowledge. In a study conducted to see gender differences in health information behaviour, men were found to be often unwilling and unmotivated to engage with health-related information.^(42)^ This can explain why women are often more knowledgeable about health-related information than men, as shown in other studies assessing the public knowledge about various health information.^(43–45)^

With the increased competition between different brands and manufacturers of energy drinks, which is reflected by various means of advertising these products targeting mainly young people, there is a need to have awareness campaigns to counteract the peer pressure and advertisements’ influence young people to decrease their consumption of energy drinks. Hence, these drinks are known for their high caffeine and sugar content, especially with the increasing number of reported caffeine toxicity cases associated with the consumption of energy drinks.^(46)^

One of the limitations of the current study is that it was based on an online self-administered questionnaire, which can cause errors of over-reporting or underreporting which affects the results to some extent. Another limitation is the sample representativeness, as most of the participants were from the middle of Jordan, where the Capital is located and most universities are, but this provides an opportunity for future similar studies to be conducted in the north and south of Jordan to see if the university students there have similar or different behaviours and perception towards energy drinks.

## Conclusion

This study is one of two cross-sectional studies conducted in Jordan to assess the knowledge, attitude, and perception of energy drinks consumption among university students in Jordan and the first to try to find a correlation between demographic characteristics and student knowledge regarding these drinks.

The prevalence of consuming energy drinks was high, and the students had neutral knowledge regarding the constituents and side effects of energy drinks. Additionally, females and those studying a major in the medical field had better knowledge. The students reported that the main reasons for consuming these drinks are helping them stay awake, study, and be more energetic.

There is a need for more structured awareness campaigns to warn students about the possible side effects of these products in an effort to reduce the consumption and popularity of energy drinks between young adults and students.

## Supporting information

Thiab et al. supplementary material Thiab et al. supplementary material

## Supplementary material

The supplementary material for this article can be found at https://doi.org/10.1017/jns.2023.90.
