# Supplementary material for: Knowledge, attitude, and perception of energy drinks consumption among university students in Jordan
Source: J Nutr Sci. 2023 Nov 3;12:e109. doi: 10.1017/jns.2023.90 (PMC10641699; doi:10.1017/jns.2023.90)
Supplement: Thiab et al. supplementary material [file S2048679023000903sup001.docx]

**Knowledge, Attitude, and Perception of Energy Drinks Consumption Among University Students in Jordan**

**الجزء الأول: المعلومات الديموغرافية**

**الجنس**

ذكر

أنثى

**العمر )بالسنوات)**

------------

**الحالة الاجتماعية**

أعزب

متزوج

مطلق

أرمل

**التخصص الدراسي الجامعي**

تخصص طبي (طب، صيدلة، تمريض، تحاليل طبية ..الخ)

تخصص علمي غير طبي (هندسة، علم حاسوب، تكنولوجيا المعلومات، علوم أساسية .. الخ)

تخصص تربية رياضية

تخصص أدبي (لغات، حقوق، أعمال، علوم إنسانية ...الخ)

**طبيعة الجامعة**

حكومية

خاصة

**سنة الدراسة**

سنة أولى

سنة ثانية

سنة ثالثة

سنة رابعة

سنة خامسة

سنة سادسة

**الشهادة الدراسية المتوقع الحصول عليها:**

دبلوم

بكالوريوس

دراسات عليا (دبلوم عالي ، ماجستير، دكتوراة)

**مكان السكن الدائم في الأردن**

العاصمة

مدينة أخرى

قرية

بادية

**دخل الأسرة بالدينار الأردني**

<250 JoD

251-500 JoD

501-750 JoD

751-1000 JoD

>1000 JoD

**التأمين الصحي**

مؤمن

غير مؤمن

**هل أنت مدخن لأي من منتجات التبغ؟** نعم – لا

**هل تعاني من أي أمراض مزمنة؟**

لا نعم، اذكرها؟.............

**الجزء الثاني: مدى المعرفة حول مشروبات الطاقة**

الرجاء الاجابة بنعم إن كنت تعتقد أن الجمل التالية صحيحة حول مشروبات الطاقة:

Knowledge score

| **مشروبات الطاقة تحتوي على مادة الكافيين** | نعم | لا |
| --- | --- | --- |
| **تحتوي بعض مشروبات الطاقة على كميات من السكر تتجاوز الحصة الطبيعية للفرد في اليوم** | نعم | لا |
| **لا تحتوي أي من مشروبات الطاقة على مستحضرات عشبية** | نعم | لا |
| **لا تحتوي أي من مشروبات الطاقة على أحماض أمينية** | نعم | لا |
| **تحتوي بعض مشروبات الطاقة على الكارنيتين الذي يزيد من حرق الدهون L-Carnitine** | نعم | لا |
| **تحتوي بعض مشروبات الطاقة على فيتامينات ومعادن** | نعم | لا |
| **تحتوي بعض مشروبات الطاقة على منتجات مشتقة من الحيوانات** | نعم | لا |
| **لا تسبب مشروبات الطاقة مشاكل في النوم** | نعم | لا |
| **من الممكن أن تسبب مشروبات الطاقة مشاكل في الجهاز الهضمي** | نعم | لا |
| **من الممكن أن تسبب مشروبات الطاقة تسارع في ضربات القلب** | نعم | لا |
| **لا يمكن أن تسبب مشروبات الطاقة صداع** | نعم | لا |
| **لا يمكن أن تزيد مشروبات الطاقة من إقدام الشخص على السلوكيات المتهورة** | نعم | لا |

**الجزء الثالث: مصادر المعلومات حول مشروبات الطاقة**

**ما هي مصادر معلوماتك عن مشروبات الطاقة؟**

المدرب الرياضي

أخصائيي التغذية

مقدمي الرعاية الصحية مثل الطبيب أو الصيدلاني

الاهل والأصدقاء

التلفاز والإذاعة

وسائل التواصل الاجتماعي

مواقع على شبكة الانترنت

غيرها، اذكرها ...................

**الجزء الرابع: استهلاك مشروبات الطاقة والتجربة المصاحبة لها**

**هل تتناول أي من مشروبات الطاقة:**

**Bison** نعم لا

**Red Bull** نعم لا

**Boom Boom** نعم لا

**Code Red**نعم لا

**لو كانت الإجابة نعم:**

**كم علبة من مشروبات الطاقة تستخدم في الأسبوع**

علبة واحدة فقط

2-3

4-5

أكثر من 5 علب

**ما هو السبب الذي يدفعك لتناول مشروبات الطاقة؟**

يساعدني في الدراسة لوقت أطول وإنجاز الواجبات

يساعدني على التركيز وبالتالي تحصيل درجات أفضل

يساعدني بالشعور بشكل أفضل ويقلل التوتروالتعب والإرهاق

السهر لوقت أكثر

لشعور بالقوة والنشاط بشكل عام

أسباب أخرى

**هل قمت بتناول أحد مشروبات الطاقة مع أدوية مثل المسكنات أو أدوية الرشح والسعال؟**

لا نعم، لطفا اذكر نوع الدواء: .....................

**هل قمت من قبل بخلط مشروب الطاقة مع فيتامينات؟**

لا نعم، مثل ماذا؟.............

**هل قمت من قبل بخلط مشروبات الطاقة مع أعشاب أو منتجات طبيعية أخرى؟**

لا نعم، مثل ماذا؟.............

**هل قمت من قبل بخلط مشروب الطاقة مع مشروبات كحولية؟**

لا نعم، مثل ماذا؟.............

**الجزء الخامس: ومدى الإدراك حول مشروبات الطاقة**

**إلى أي مدى تتفق أو تختلف مع الجمل التالية:**

| **مشروبات الطاقة مفيدة للصحة** | موافق بشدة | موافق | محايد | غير موافق | غير موافق بشدة |
| --- | --- | --- | --- | --- | --- |
| **مشروبات الطاقة تزيد القدرة على التحمل** | موافق بشدة | موافق | محايد | غير موافق | غير موافق بشدة |
| **مشروبات الطاقة تزيد القوة** | موافق بشدة | موافق | محايد | غير موافق | غير موافق بشدة |
| **مشروبات الطاقة تزيد النشاط** | موافق بشدة | موافق | محايد | غير موافق | غير موافق بشدة |
| **مشروبات الطاقة تحسن من الأداء الرياضي أو الوظيفي** | موافق بشدة | موافق | محايد | غير موافق | غير موافق بشدة |
| **مشروبات الطاقة تقلل الشعور بالألم** | موافق بشدة | موافق | محايد | غير موافق | غير موافق بشدة |
| **مشروبات الطاقة ليس لها أعراض جانبية** | موافق بشدة | موافق | محايد | غير موافق | غير موافق بشدة |

**Knowledge, Attitude, and Perception of Energy Drinks Consumption Among University Students in Jordan**

**Part One: Demographic Information**

**Gender**

Male

Female

**Age (in years)**

------------

**Marital status**

Single

Married

Divorced

Widowed

**University academic major**

Medical (medicine, pharmacy, nursing, medical tests, etc.)

Non-medical science major (engineering, computer science, information technology, basic sciences, etc.)

Sport pedagogy

literature major (languages, law, business, humanities, etc.)

**University Sector**

Governmental

Private

**Year of study**

First year

Second Year

Third year

Fourth year

Fifth year

Sixth year

**Academic certificate expected to be obtained**

Diploma

BSc

Postgraduate studies (higher diploma, MSc, Phd)

**Permanent place of residence in Jordan**

The Capital, Amman

Another city

A village

Badia

**Family income in Jordanian dinars**

<250 joD

251-500 JoD

501-750 JOD

751-1000 JoD

>1000 JoD

**Health insurance**

Insured

**Are you a smoker of any tobacco products?** Yes No

**Do you suffer from any chronic diseases?**

No

Yes, mention it?

**Part 2: Extent of knowledge about energy drinks**

**Please answer yes if you think the following statements are true about energy drinks: (Knowledge score)**

1. Energy drinks contain caffeine. Yes No
2. Some energy drinks contain more sugar than the normal quota per person per day. Yes No
3. None of the energy drinks contain herbal preparations. Yes No
4. None of the energy drinks contain amino acids. Yes No
5. Some energy drinks contain L-Carnitine, which increases fat burning. Yes No
6. Some energy drinks contain vitamins and minerals. Yes No
7. Some energy drinks contain animal-derived products. Yes No
8. Energy drinks don't cause sleep problems. Yes No
9. Energy drinks can cause digestive problems. Yes No
10. Energy drinks can cause a rapid heartbeat. Yes No
11. Energy drinks can't cause headaches. Yes No
12. Energy drinks cannot increase a person's impulsive behavior. Yes No

**Part 3: Sources of information about energy drinks**

**What are your sources of information about energy drinks?**

Sports coaches

Nutritionists

Health care providers such as a doctors or pharmacists

Family and friends

TV and radio

Social media

Websites

Others, mention them…………………………………

**Part Four: Energy Drink Consumption and Experience**

**Do you drink any of the energy drinks?**

Bison: yes no

Red Bull: Yes No

Boom Boom: Yes No

Code Red: Yes No

**If the answer is yes: How many cans of energy drinks do you use per week?**

only one can

2-3

4-5

More than 5 cans

**What is the reason that drives you to drink energy drinks?**

It helps me study for a longer time and accomplish tasks such as homework

It helps me focus and thus get better grades

It helps me feel better and reduces stress, fatigue and exhaustion

To stay awake for a longer time

To feel strong and energetic in general

Other reasons

**Have you taken an energy drink with medications such as painkillers or cough and cold remedies?**

No Yes, please indicate the type of medicine: …………………………………………………

**Have you ever mixed an energy drink with vitamins?**

No yes, like what?.............

**Have you ever mixed energy drinks with other herbs or natural products?**

No yes, like what?.............

**Have you ever mixed energy drink with alcoholic drinks?**

No yes, like what?.............

**Part Five: The Perception of Energy Drinks**

**To what extent do you agree or disagree with the following statements:**

**Energy drinks are good for health:** Strongly Agree Neutral Disagree Strongly disagree

**Energy drinks increase stamina:** Strongly Agree Neutral Disagree Strongly disagree

**Energy drinks increase strength:** Strongly Agree Neutral Disagree Strongly disagree

**Energy drinks increase activity:** Strongly Agree Neutral Disagree Strongly disagree

**Energy drinks improve athletic or functional performance:** Strongly Agree Neutral Disagree Strongly disagree

**Energy drinks reduce pain:** Strongly Agree Neutral Disagree Strongly disagree

**Energy drinks have no side effects:** Strongly Agree Neutral Disagree Strongly disagree
